# Supplementary material for: Circulating Microparticles Are Differentially Increased in Lowlanders and Highlanders with High Altitude Induced Pulmonary Hypertension during the Cold Season
Source: Cells. 2022 Sep 20;11(19):2932. doi: 10.3390/cells11192932 (PMC9563667; doi:10.3390/cells11192932)
Supplement: Supplementary file 1 [file cells-11-02932-s001.zip › cells-1897473-supplementary.pdf]

SUPPLEMENTAL DATA

Circulating microparticles and cold are active players in the pathology of high altitude induced pulmonary hypertension in Kyrgyz highlanders

Akylbek Sydykov, Aleksandar Petrovic, Abdirashit M. Maripov, Marija Gredic, Daniel Gerd Bermes, Nadira Kushubakova, Kubatbek Muratali Uulu, Christina Vroom, Meerim Cholponbaeva, Melis Duishobaev, Samatbek Satybaldyev, Nurgul Satieva, Argen Mamazhakypov, Meerim Sartmyrzaeva, Nazgul Omurzakova, Zhainagul Kerimbekova, Nursultan Baktybek, Cholpon Kulchoroeva, Oleg Pak, Lan Zhao, Norbert Weissmann, Sergey Avdeev, Leonid N. Maslov, Hossein Ardeschir Ghofrani, Ralph Theo Schermuly, Akpay S. Sarybaev, Djuro Kosanovic

Methods

Flow cytometry characterization and quantification of endothelial and different inflammatory cells-derived MPs

Briefly, 10 µl of PFP was re-suspended in 0.5% BSA (Sigma), 0.2 mM EDTA (Roth) HBSS (Gibco) without Ca^{2+} and Mg^{2+} and stained with single fluorochrome-conjugated antibody or respective IgG control. After centrifugation at 19800 g, MPs were re-suspended in the same buffer and analyzed using S3E cell sorter (Biorad). Data were analyzed using FlowJo Vx (TreeStar). The instrument calibration was controlled daily using Proline™ Universal Calibration Beads (Biorad). Calibration of the instrument for the MPs analysis was performed using Megamix-Plus FSC beads (diameters 0.1-0.9 µm) (BioCytex). For characterization of the MPs, following antibodies were used: Alexa Fluor 488 mouse anti-human CD68 (macrophages) (R&D Systems), PECy5™ mouse anti-human CD3 (T-cells) (BD Pharmingen),

PECy5™ mouse anti-human CD62E (endothelial cells) (BD Pharmingen) and the corresponding IgG controls. Results were expressed as events per µl of PFP.

Effects of isolated MPs on proliferation and apoptosis of human pulmonary artery smooth muscle cells (PASMCs)

Human PASMCs (7500 cells per well) were seeded in 96-well black-walled plate and pre-incubated for 48 hours at 37°C in water saturated incubators with 10µg/ml of isolated MPs or NaCl control. Following the pre-incubation period, pSIVA-IANBD fluorescent probe was added and plate was transferred to IncuCyte live cell fluorescence microscopy imaging system for 6h. Images were taken every 45 minutes and analyzed by IncuCyte ZOOM software. Obtained percentage values of green object confluence were normalized to cell surface confluence. Proliferation of human PASMCs was determined by Cell Proliferation ELISA, 5-bromo-2'-deoxyuridine (BrdU) assay (Sigma Aldrich), following the manufacturer instructions. In more details, 7500 cells per well were seeded into 24-well plates and following the 24 hours starvation period PASMCs were incubated for 48 hours in Smooth Muscle Basal Medium in the presence of 10µg/ml of isolated MPs or NaCl control. The absorbance of the substrate reaction was measured at 370 nm (reference wavelength 492 nm).

The contents of different microRNAs (miRNAs) in isolated MPs: quantitative real-time polymerase chain reaction (qRT-PCR)

Isolated MPs were lyzed by addition of 700 µl of QIAzol lysis buffer and total RNA was isolated using the miRNeasy Mini Kit (Qiagen), according to the manufacturer

protocol. The amount of recovered RNA was quantified by Nanodrop (Thermo Fisher Scientific).

Expression levels of selected miRNAs: miRNA-210, miRNA-145, miRNA-17 and miRNA-133a-3p were assessed by qRT-PCR and TaqMan miRNA assays (Thermo Fisher Scientific). 10 ng of total RNA was reversely transcribed to cDNA by miRNA specific looped RT primers using TaqMan miRNA reverse transcription kit (Thermo Fisher Scientific). 2 µl of cDNA template was added to 10 µl TaqMan Universal Master Mix II (Thermo Fisher Scientific), 1 µl TaqMan small RNA assay (PCR primer set and miRNA specific TaqMan probe) and 7 µl nuclease free water. All samples were run in duplicates. PCR reaction was performed using CFX96 PCR detection system (Bio-Rad). Ct values were calculated with logarithmic fluorescence signal and a threshold value of 0.1. Delta Ct method was used to quantify relative miRNA expression. The relative amount of miRNA was normalized to the endogenous miRNA-425-5p as a reference.
